# Oncogenic AKT1(E17K) mutation induces mammary hyperplasia but prevents HER2-driven tumorigenesis

**DOI:** 10.18632/oncotarget.8191

**Published:** 2016-03-18

**Authors:** Maria L. Mancini, Evan C. Lien, Alex Toker

**Affiliations:** ^1^ Department of Pathology and Cancer Center, Beth Israel Deaconess Medical Center, Harvard Medical School, Boston, MA, USA; ^2^ Champions Oncology, Science and Technology Park at Johns Hopkins, Baltimore, MD, USA

**Keywords:** Akt, breast cancer, PI 3-kinase, HER2, estrogen receptor

## Abstract

One of the most frequently deregulated signaling pathways in breast cancer is the PI 3-K/Akt cascade. Genetic lesions are commonly found in *PIK3CA*, *PTEN*, and *AKT*, which lead to excessive and constitutive activation of Akt and downstream signaling that results in uncontrolled proliferation and increased cellular survival. One such genetic lesion is the somatic *AKT1(E17K)* mutation, which has been identified in 4-8% of breast cancer patients. To determine how this mutation contributes to mammary tumorigenesis, we constructed a genetically engineered mouse model that conditionally expresses human *AKT1(E17K)* in the mammary epithelium. Although AKT1(E17K) is only weakly constitutively active and does not promote proliferation *in vitro*, it is capable of escaping negative feedback inhibition to exhibit sustained signaling dynamics *in vitro*. Consistently, both virgin and multiparous *AKT1(E17K)* mice develop mammary gland hyperplasia that do not progress to carcinoma. This hyperplasia is accompanied by increased estrogen receptor expression, although exposure of the mice to estrogen does not promote tumor development. Moreover, *AKT1(E17K)* prevents *HER2-*driven mammary tumor formation, in part through negative feedback inhibition of RTK signaling. Analysis of TCGA breast cancer data revealed that the mRNA expression, total protein levels, and phosphorylation of various RTKs are decreased in human tumors harboring *AKT1(E17K)*.

## INTRODUCTION

One of the most frequently deregulated pathways in human cancers is the phosphoinositide 3-kinase (PI 3-K) and Akt signaling cascade [1]. This is particularly evident in breast cancer where mutations exist in virtually all of the proteins that lead to activation of PI 3-K and its downstream effectors. Activation of cell surface receptors, particularly receptor tyrosine kinases (RTKs), leads to activation of class I PI 3-K, which catalyzes the synthesis of PtdIns-3,4,5-P3 (PIP3) from PtdIns-4,5-P2 (PIP2). PIP3 functions as a true second messenger by recruiting multiple effector molecules, one of which is the Akt/PKB protein kinase that directly binds to PIP3 through its pleckstrin homology (PH) domain. This binding facilitates the phosphorylation of Akt at Thr308 and Ser473 mediated by phosphoinositide-dependent kinase-1 (PDK-1) and mammalian target of rapamycin complex 2 (mTORC2), respectively [2]. Finally, activated Akt translocates to multiple cellular compartments where it phosphorylates a large number of substrates that transduce the signal to a variety of cellular responses that are intimately associated with malignancy, including cell proliferation, growth, motility, survival and metabolic reprogramming [3]. The Akt family comprises three isoforms - AKT1 (PKBα), AKT2 (PKBβ), and AKT3 (PKBγ) - which have non-redundant functions in various physiological as well as pathophysiological conditions [4].

The most frequent genetic lesions in this pathway comprise oncogenic mutations in *PIK3CA*, the gene that encodes the p110α isoform of class I PI 3-K [5]. Other breast cancer mutations prevalent in this pathway include mutational or epigenetic inactivation of Phosphatase and Tensin Homolog (*PTEN*), a lipid phosphatase that terminates PI 3-K signaling by dephosphorylating PIP3. Both oncogenic *PIK3CA* mutations and inactivation of *PTEN* lead to excessive and constitutive activation of Akt and downstream signaling, resulting in uncontrolled proliferation and increased cellular survival [6]. Mutations and amplifications in the Akt genes themselves have also been identified in various human solid tumors. In the context of breast cancer, the *AKT1(E17K)* somatic mutation was first identified in breast cancer but is also found in lung, bladder, endometrial, urothelial and prostate cancers [7-13]. The frequency of the *AKT1(E17K)* mutation in breast cancers ranges from 4-8%. This oncogenic mutation renders Akt constitutively active by broadening the lipid specificity of the Akt PH domain [14], thus enabling its transforming capacity in fibroblasts *in vitro* and leukemias *in vivo [7]*. Interestingly, in breast cancer *AKT1(E17K)* is mutually exclusive with *PIK3CA* and *PTEN* mutations [15], although in other cancers such as endometrial carcinoma, these mutations frequently co-exist in the same tumor [12]. Furthermore, *AKT1(E17K)* has been found predominantly in estrogen receptor (ER)-positive breast tumors [16]. However, *in vitro* studies have provided inconclusive information regarding the functional advantages this oncogenic mutation confers [17]. Expression of AKT1(E17K) has been shown to enhance cell migration and resistance to chemotherapeutic agents in luminal breast cancer cells [17, 18]. Similarly, knock-in of the *AKT1(E17K)* mutation into MCF-7 ER-positive cells in which oncogenic *PIK3CA(E545K)* has been restored to the wild-type allele restores proliferation and tumor growth *in vivo*, arguing that at least in ER-positive luminal breast cancer cells, *AKT1(E17K)* can function as a *bona fide* oncogene [19]. It is also worth noting that an analogous E17K mutation has been identified in *AKT2* in one breast cancer patient [20] and in *AKT3* in melanomas [21]. Moreover, a recurrent MAGI3-Akt3 fusion protein that results in a truncated form of the *MAGI3* gene fused in frame to *AKT3* at the E17 residue of Akt3 has been identified in breast cancers [22]. The mechanisms by which any of these somatic mutations contribute to malignancy have yet to be reported.

To date, no studies have examined the capacity of *AKT1(E17K)* to drive mammary cancer in a genetically engineered mouse model. Previous studies have addressed the contribution of AKT1 activity to mammary tumorigenesis using constitutively active AKT1 transgenes driven by the mouse mammary tumor virus (MMTV) promoter. MMTV-MyrAKT1 mice treated with DMBA to induce chemical carcinogenesis develop ER-positive mammary tumors [23]. In addition, transgenic mice harboring a phospho-mimetic *AKT1(T308D/S473D)* mutant in combination with *HER2* display a decrease in tumor latency and accelerated tumor growth, but decreased incidence of metastases, consistent with AKT1 functioning as a metastasis suppressor [24, 25]. Studies using AKT1 and AKT2 knockout mice have arrived at similar conclusions [26].

Since any association between AKT1 and ER has not been explored *in vivo,* and there are no models to evaluate the contribution of *AKT1(E17K)* to mammary tumorigenesis, we generated a mammary-specific inducible *AKT1(E17K)* transgenic mouse. We present evidence indicating that *AKT1(E17K)* is not sufficient for transformation *in vivo*, but induces mammary gland hyperplasia in both virgin and multiparous females. In addition, combination of MMTV-driven *AKT1(E17K)* with MMTV-*HER2* overexpression prevents *HER2-*driven mammary tumor formation, in part through negative feedback inhibition of RTK signaling mediated by AKT1(E17K).

## RESULTS

### AKT1(E17K) escapes negative feedback inhibition but does not enhance proliferation of mammary epithelial cells *in vitro*

**Figure 1:**
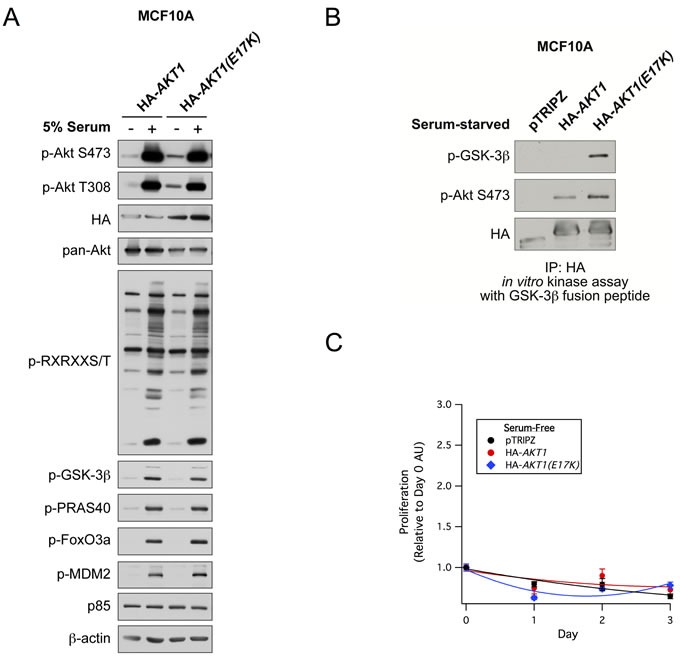
AKT1(E17K) has weak basal constitutive activity and does not promote proliferation in MCF10A cells **A.** MCF10A cells expressing tet-on HA-*AKT1*/pTRIPZ or HA-*AKT1(E17K)*/pTRIPZ were treated with 150 ng/ml doxycycline for 48 h to induce *AKT1* or *AKT1(E17K)* expression. Cells were serum-starved for 16 h and then treated with 5% serum for 10 min. Whole cell lysates were subjected to immunoblotting. **B.** Anti-HA immunoprecipitates from serum-starved cells described above were used in *in vitro* kinase assays with a GSK-3β fusion peptide. The kinase reaction was terminated and samples were immunoblotted with the indicated antibodies. **C.** MCF10A cells described above were grown in the absence of serum and growth factors in media maintained with 150 ng/ml doxycycline. Cell proliferation on days 0, 1, 2, and 3 were measured with the WST-1 assay, and values are expressed relative to day 0.

To study the functional significance of the *AKT1(E17K)* mutation in breast cancer, we developed a system to stably express either wild-type *AKT1* or *AKT1(E17K)* in a doxycycline-inducible manner in the non-tumorigenic immortalized MCF10A breast epithelial cell line. Cells were serum-starved overnight and stimulated with 5% serum. Consistent with previous studies [17], basal phosphorylation of AKT1(E17K) at Ser473 and Thr308 is moderately elevated compared to wild-type AKT1 (Figure 1A). However, this does not translate into significantly enhanced phosphorylation of downstream Akt substrates as measured with a substrate-directed Akt motif antibody, as well as antibodies against known Akt substrates (Figure 1A). This is despite the fact that in a cell-free system, isolated AKT1(E17K) has significantly elevated protein kinase activity toward the model substrate GSK-3β, again when compared to wild-type AKT1. Apparently, this enhanced intrinsic kinase activity is not sufficient to propagate signals to constitutive downstream substrate phosphorylation in the absence of stimuli. Consistently, AKT1(E17K) cannot promote the proliferation of cells in the absence of serum and growth factors (Figure 1C), nor does it provide a proliferative advantage in full growth media (data not shown).

**Figure 2:**
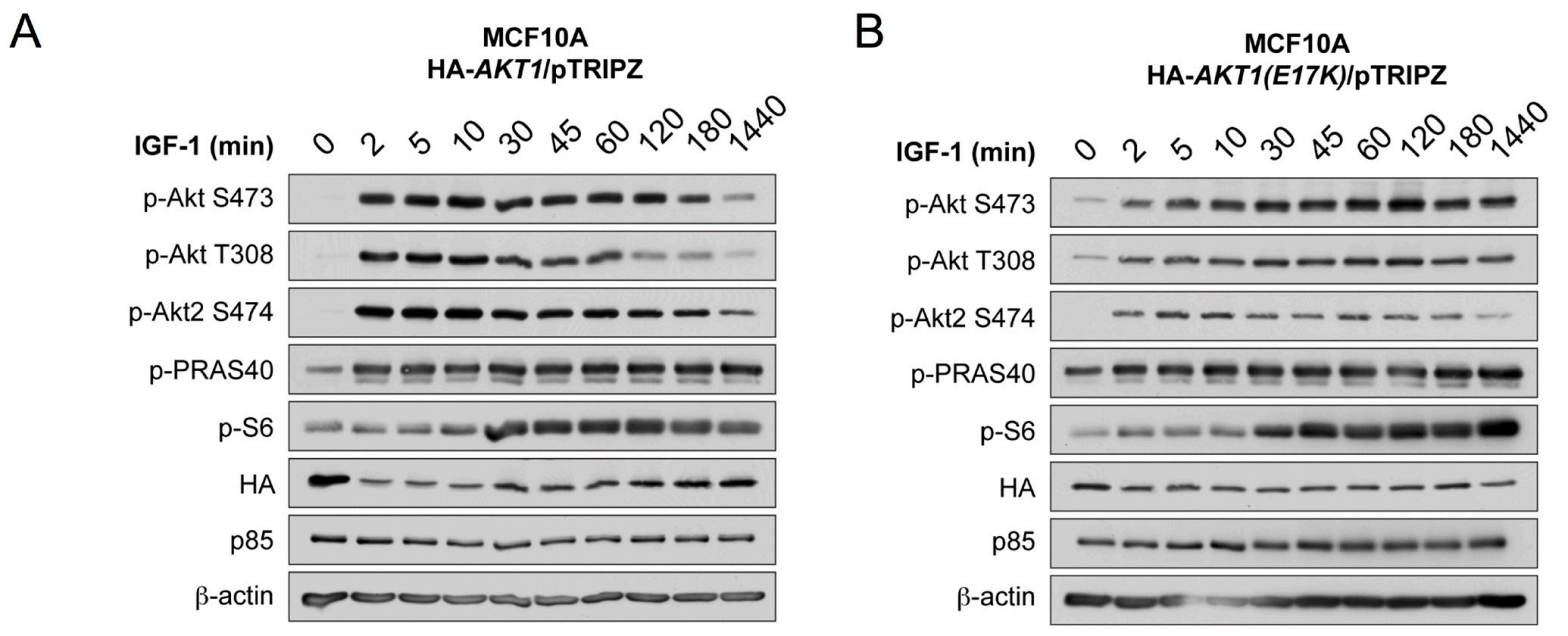
AKT1(E17K) escapes negative feedback inhibition to exhibit sustained activation kinetics in response to IGF-1 MCF10A cells expressing tet-on HA-*AKT1*/pTRIPZ **A.** or HA-*AKT1(E17K)*/pTRIPZ **B.** were treated with 150 ng/ml doxycycline for 48 h to induce *AKT1* or *AKT1(E17K)* expression. Cells were serum-starved for 16 h and then treated with 100 μg/ml IGF-1 for the indicated times. Whole cell lysates were subjected to immunoblotting.

Since multiple feedback loops exist in the PI 3-K and Akt pathway to maintain homeostatic control, and oncogenic mutations in genes that modulate this pathway can often escape this feedback regulation, we next evaluated the kinetics of AKT1(E17K) activation in MCF10A cells. Cells expressing wild-type AKT1 show a robust induction of Akt phosphorylation in response to IGF-1 as early as 2 min, translating into Akt substrate phosphorylation (pPRAS40). This activation attenuates by 1 h post-stimulation and returns to basal levels by 24 h (Figure 2A), as would be expected by feedback inhibition. By contrast, cells expressing AKT1(E17K) show sustained Akt activation (pSer473, pThr308, pPRAS40) out to 24 h post-stimulation (Figure 2B). The kinetics of endogenous Akt2 phosphorylation are still subject to feedback inhibition as judged by phosphorylation of pSer474 in both wild type and AKT1(E17K) expressing cells. This indicates that AKT1(E17K) specifically escapes feedback inhibition, allowing for sustained signal propagation.

### AKT1(E17K) transgenic mice develop mammary hyperplasia associated with increased estrogen receptor expression

**Figure 3:**
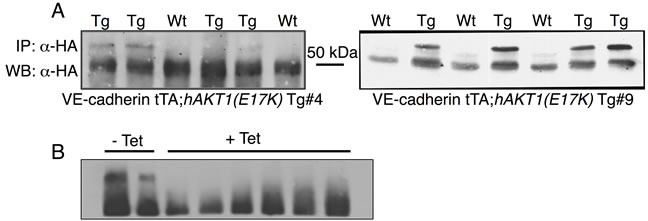
Generation of the pTET;*hAKT1(E17K)* transgenic mice The *hAKT1(E17K)* founder lines were selected based on transgene expression in the newborn liver from VE-Cadherin-tTA;*hAKT1(E17K)* mice by IP/Western using human α-HA **A.** and after exposure to tetracycline **B.**

The above data demonstrate that the *AKT1(E17K)* mutation is not sufficient to drive proliferation in non-tumorigenic breast epithelial cells *in vitro*. However, MCF10A cells do not express estrogen receptor, and the *AKT1(E17K)* mutation is strongly associated with ER-positive tumors in patients [16]. Furthermore, the majority of ER-positive breast cancer cell lines also harbor *PIK3CA* or *PTEN* mutations, which are mutually exclusive with *AKT1(E17K)* [15]. Therefore, to evaluate the contribution of *AKT1(E17K)* to mammary tumorigenesis in the context of ER expression, we generated a tissue-specific, *AKT1(E17K)* transgenic mouse line by cloning human HA-*AKT1(E17K)* (*hAKT1(E17K)*) into the tetracycline-responsive (tet-off) pTET splice vector. Two founder lines (Tg #4 and Tg #9) were selected based on protein expression by crossing candidate founders determined by PCR with a VE-Cadherin-tTA driver mouse for constitutive expression of the transgene (Figure 3A). This allowed us to perform expression analysis on livers harvested from newborn pups to expedite the initial screening of protein levels among founder lines. To validate the responsiveness to tetracycline, we added tetracycline to the drinking water of VE-Cadherin-tTA;*hAKT1(E17K)* mice to turn off protein expression (Figure 3B). Upon selection of two *hAKT1(E17K)* expressing lines, we validated activity of the transgene in the mammary epithelium using MMTV-tTA driver mice, revealing expression of the transgene using anti-HA IHC in control MMTV-tTA or MMTV-tTA;*hAKT1(E17K)* lines (data not shown).

**Figure 4:**
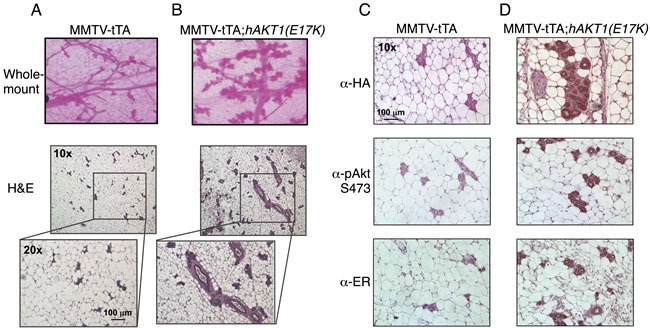
Virgin MMTV-tTA;*hAKT1(E17K)* females exhibit mammary gland hyperplasia with an increase in ER expression Wholemount analysis of mammary glands was performed from MMTV-tTA **A.** or MMTV-tTA;*hAKT1(E17K)* **B.** mice. Hematoxylin and eosin staining was also done on sections of paraffin embedded mammary glands from MMTV-tTA (A) or MMTV-tTA;*hAKT1(E17K)* (B) mice. Immunohistochemistry with α-HA, α-pAkt Ser473, and α-ER antibodies was performed for paraffin embedded mammary glands from MMTV-tTA **C.** or MMTV-tTA;*hAKT1(E17K)* **D.** mice. All panels are representative of analyses of at least *n* = 5 mice.

To address whether *AKT1(E17K)* is sufficient to drive oncogenic signaling in the mammary epithelium, we initially assessed transformation in virgin female transgenic mice. MMTV-tTA;*hAKT1(E17K)* mice were monitored for the presence of palpable mammary tumors over the course of 1 year of constitutive transgene expression. No palpable tumors were detected in any of the virgin females. Whole mount and histological analyses of mammary glands confirmed the absence of tumors as a result of prolonged MMTV-driven *hAKT1(E17K)* expression, compared to control mice harboring MMTV alone (Figure 4A and 4B). However, all mammary glands of transgenic mice demonstrated marked acinar hyperplasia beginning at 6 months of age (Figure 4B). Immunohistochemical analysis revealed that the hyperplasia results from an expansion of the myoepithelium, since the hyperplastic mammary acini are negative for CK8, a luminal marker, but maintain expression of CK14, a myoepithelial marker (Supplementary Figure S1). Furthermore, expression of the *hAKT1(E17K)* transgene results in increased expression of ER (Figure 4D, bottom panel), concomitant with expression of the transgene as revealed by staining for anti-HA and anti-pSer473 (Figure 4D, top and middle panels), again compared to control mice (Figure 4C). This is consistent with published data showing that the *AKT1(E17K)* somatic mutation is associated with ER-positive breast tumors [16].

**Figure 5:**
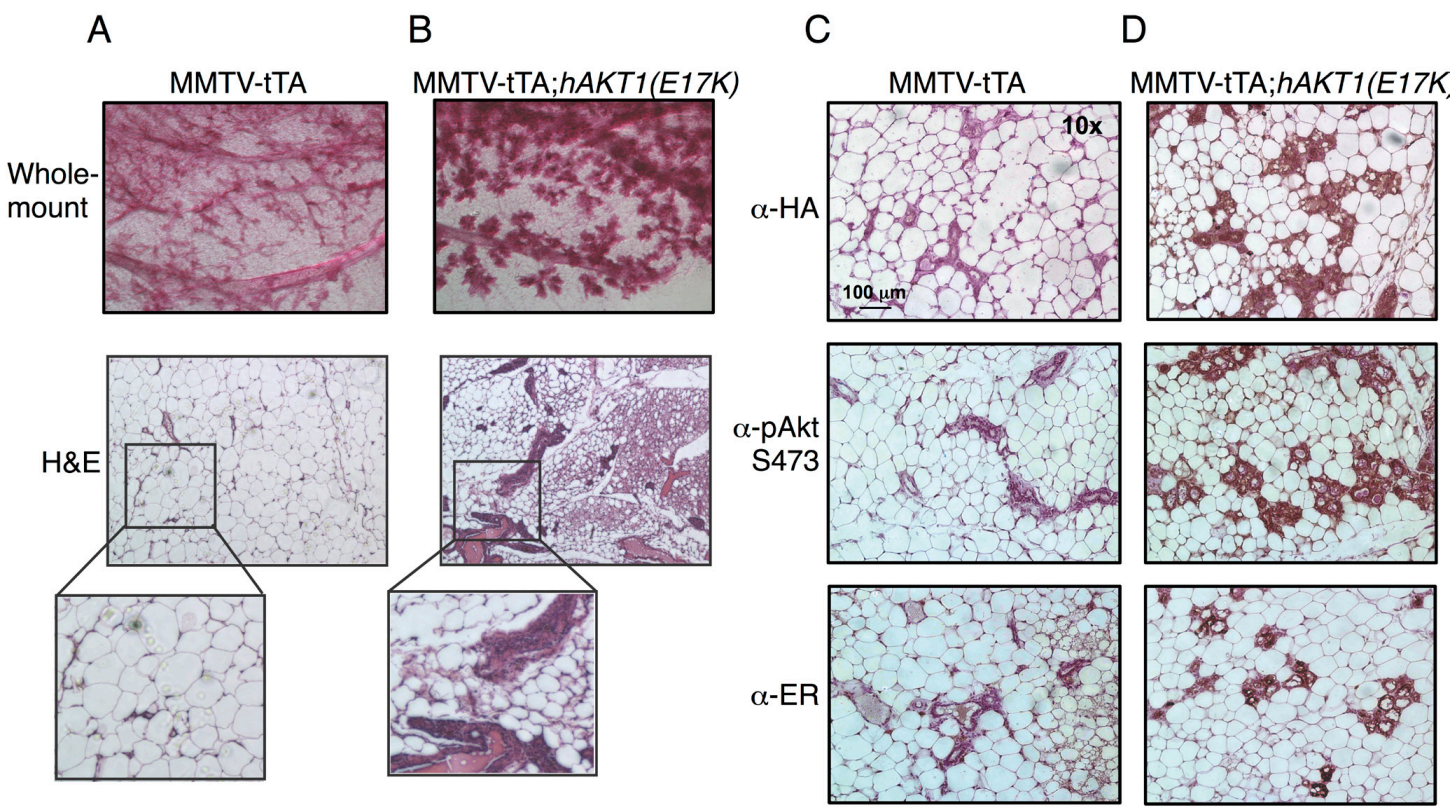
Multiparous MMTV-tTA;*hAKT1(E17K)* females exhibit mammary gland hyperplasia with an increase in ER expression Wholemount analysis of mammary glands was performed from MMTV-tTA **A.** or MMTV-tTA;*hAKT1(E17K)* **B.** mice. Hematoxylin and eosin staining was also done on sections of paraffin embedded mammary glands from MMTV-tTA (A) or MMTV-tTA;*hAKT1(E17K)* (B) mice. Immunohistochemistry with α-HA, α-pAkt Ser473, and α-ER antibodies was performed for paraffin embedded mammary glands from MMTV-tTA **C.** or MMTV-tTA;*hAKT1(E17K)* **D.** mice. All panels are representative of analyses of at least *n* = 5 mice.

We next determined whether signaling events contributing to the proliferation and morphogenesis of the mammary gland as a result of pregnancy provide the necessary factors to promote transformation. MMTV-tTA;*hAKT1(E17K)* mice were paired multiple times and monitored for 1 year for evidence of palpable tumors. Similar to virgin females, whole mount and histological analyses confirmed the absence of tumors. However, multiparous females display a more dramatic mammary gland hyperplasia at 1 year compared to virgin females and control MMTV mice (Figure 5A and 5B). In addition, expression of *hAKT1(E17K)* also leads to increased ER expression in hyperplastic mammary acini compared to control (Figure 5C and Figure 5D, bottom panel). Taken together, these data demonstrate that constitutive expression of *hAKT1(E17K)* causes mammary hyperplasia in both virgin and multiparous females with an associated increase in ER expression.

We also determined if mammary hyperplasia occurs as a result of *hAKT1(E17K)* signaling during early events in mammary gland development. Tetracycline was added to the drinking water to turn off transgene expression just after the onset of puberty in virgin females at approximately 6-7 weeks of age. Mice were administered tetracycline for a total of 6 months. Mammary glands analyzed by whole mount and histological analysis appear normal, and hyperplasia is not observed (data not shown). These data indicate that the events driving mammary hyperplasia in MMTV-tTA;*hAKT1(E17K)* transgenic animals do not occur as a result of alterations in early mammary developmental programs.

### Estrogen does not enable *AKT1(E17K)* to promote mammary tumorigenesis

Numerous studies have indicated multiple interactions between PI 3-K/Akt signaling and ER function in breast cancer. Growth factor signaling promotes ER phosphorylation, which alters receptor conformation, affinity and transcriptional activity [27]. ERα can also directly bind the p85 subunit of PI 3-K, allowing estrogen stimulation to potentiate PI 3-K activity leading to Akt activation [28]. Moreover, overexpression of HER2 simultaneously with ERα in breast cancer modulates endocrine resistance [29]. Stimulation of mammary epithelial cells with estradiol results in increased expression and activity of the estrogen receptor [30], and prolonged exposure to estrogen accelerates mammary transformation in mice [31]. Since the *AKT1(E17K)* somatic mutation is only detected in patients with ER-positive breast tumors [16], we evaluated whether exposure to estradiol accelerates the MMTV-tTA;*hAKT1(E17K)* mammary gland phenotype. Multiparous MMTV-tTA;*hAKT1(E17K)* transgenic mice were exposed to a slow-release pellet of 17β-estradiol for 63 days. Whole mount and histological analyses revealed that prolonged exposure to 17β-estradiol results in an arrested state of lactation in both MMTV-tTA;*hAKT1(E17K)* and MMTV-tTA control mice (Supplementary Figure S2A and S2B). We were unable to detect mammary tumors in either MMTV-tTA;*hAKT1(E17K)* or MMTV-tTA control mice; however, hyperplasia was observed in MMTV-tTA;*hAKT1(E17K)* mice (Supplementary Figure S2C and S2D). Interestingly, increased pAkt Ser473 staining is observed as a result of estradiol stimulation in the acini of MMTV-tTA control mammary glands, with a further increase in MMTV-tTA;*hAKT1(E17K)* mice (Figure 4C and 4D). As expected, elevated expression of ER is also observed in both transgenic and control mammary glands as a result of prolonged estradiol stimulation. Taken together, these results further support a role for *AKT1(E17K)* in mediating upstream and downstream ER signaling.

### *AKT1(E17K)* suppresses *HER2*-mediated mammary tumorigenesis

**Figure 6:**
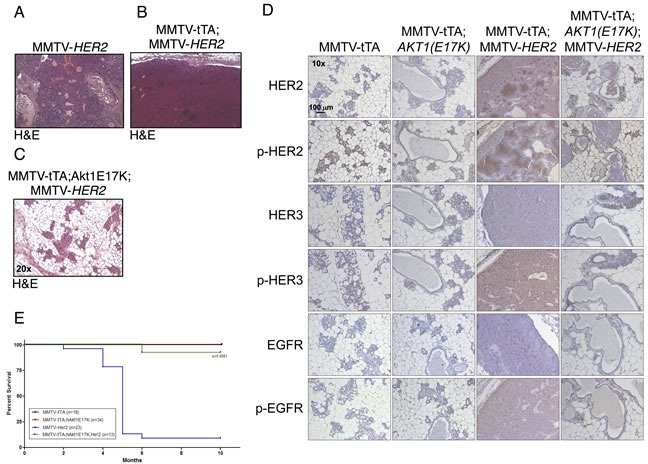
MMTV-tTA;*hAKT1(E17K)* expression in the mammary epithelium prevents MMTV-HER2 driven tumor formation with an associated decrease in EGFR family expression and phosphorylation H&E staining on paraffin sections of mammary glands from MMTV-tTA;*hAKT1(E17K)* mice crossed with MMTV-*HER2* homozygous females. **A.** MMTV-*HER2*, **B.** MMTV-tTA;MMTV-*HER2*, or **C.** MMTV-tTA;hAKT1(E17K);MMTV-*HER2* transgenic mice. **D.** Immunostaining against α-HER2, α-pHER2 (pTyr877), α-HER3, α-pHER3 (pTyr1289), α-EGFR and α-pEGFR (pTyr1068) on paraffin sections of mammary glands from MMTV-tTA;*hAKT1(E17K)* mice crossed with MMTV-*HER2* homozygous females. **E.** Kaplan-Meier survival curves were generated for all groups. A log-rank (Mantel Cox) test was used to determine statistical significance. Number of mice used for the analysis are listed in the box inset. All panels are representative of analyses of at least *n* = 5 mice.

Integrative genomic studies have indicated that the *AKT1(E17K)* mutation in breast cancer is mutually exclusive with *HER2* [16]. However, transgenic expression of activated phospho-mimetic or myristoylated AKT1 mutants in the mammary gland in combination with MMTV-*HER2* significantly decreases tumor latency, suggesting that signaling through Akt can enhance the effects of *HER2* [25, 32]. By contrast, we find that tumor formation in double MMTV-tTA;*hAKT1(E17K)*;MMTV-*HER2* transgenic mice is completely abolished compared to MMTV-*HER2* alone (Figure 6A-6C). However, the MMTV-tTA;*hAKT1(E17K)*;MMTV-*HER2* mammary glands display mammary gland hyperplasia similar to that observed in MMTV-tTA;*hAKT1(E17K)* transgenic mice (Figure 6C, compared to Figure 4B). MMTV-tTA;*hAKT1(E17K)*;MMTV-*HER2* mice exposed to tetracycline (to turn off transgene expression) in the drinking water develop mammary tumors as expected (data not shown).

A previous study has shown that expression of an activated Akt allele in a *HER2*-driven background in the mouse mammary gland leads to decreased expression and phosphorylation of a number of RTKs, including HER3 and EGFR [32]. Consistent with this, we find that MMTV-tTA;*hAKT1(E17K)*;MMTV-*HER2* mice express significantly lower total and tyrosine-phosphorylated EGFR, HER2 and HER3, to the same extent that is observed in MMTV-tTA;*hAKT1(E17K)* mice (Figure 6D). Moreover, MMTV-tTA;*hAKT1(E17K)*;MMTV-*HER2* mice show significantly improved survival to the same extent that is seen in control or MMTV-tTA;*hAKT1(E17K)* mice, when compared to MMTV-*HER2* mice (Figure 6E). These data are consistent with AKT1(E17K) attenuating RTK signaling through feedback inhibition.

**Figure 7:**
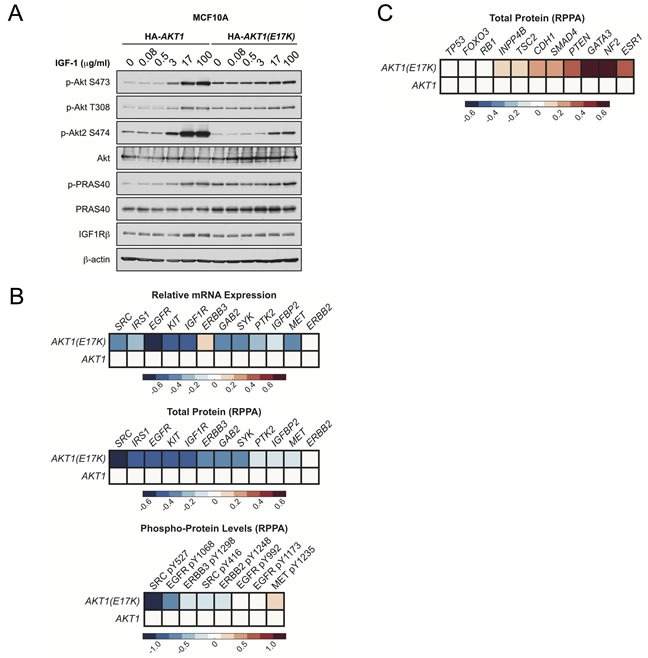
AKT1(E17K) suppresses RTK expression and phosphorylation through negative feedback inhibition **A.** MCF10A cells expressing tet-on HA-*AKT1*/pTRIPZ or HA-*AKT1(E17K)*/pTRIPZ were treated with 150 ng/ml doxycycline for 48 h to induce *AKT1* or *AKT1(E17K)* expression. Cells were serum-starved for 16 h and then treated with the indicated concentrations of IGF-1 for 10 min. Whole cell lysates were subjected to immunoblotting. **B.**-**C.** Data for mRNA, RPPA total protein and RPPA phospho-protein was downloaded from the TCGA data set on the cBIO Portal (http://www.cbioportal.org/public-portal/). Heat maps were generated using MatLab and represent microarray/RPPA values for the indicated genes/proteins, which are expressed relative to values in the wild-type AKT1 group.

To determine if AKT1(E17K) controls negative feedback, we examined dose-dependent activation of endogenous Akt2 in MCF10A cells expressing AKT1(E17K). As predicted, activation of endogenous Akt2 is suppressed by AKT1(E17K) expression in cells stimulated with IGF-1, compared to cells expressing wild-type AKT1 (Figure 7A). No changes in the total levels of AKT or IGF-1-1R are observed. Finally, we analyzed mRNA expression and reverse-phase protein array (RPPA) data from the breast cancer TCGA data set for tumors harboring *AKT1(E17K)* [33]. Consistent with the findings from the MMTV-tTA;*hAKT1(E17K)*;MMTV-*HER2* mice, analysis of RTKs and RTK-related proteins in the microarray and RPPA data sets reveals that a significant proportion of these show a downward trend in mRNA expression, protein levels, and phosphorylation, although this does not reach statistical significance due to the relatively small number of *AKT1(E17K)* cases (Figure 7B). Moreover, total ER (*ESR1*) is elevated in *AKT1(E17K)* tumors (Figure 7C), consistent with elevated ER in the MMTV-tTA;*hAKT1(E17K)* mice (Figure 4D). Intriguingly, we also note a significant up-regulation of several tumor suppressors in *AKT1(E17K)* tumors, including neurofibromin 2 (*NF2*), *PTEN* and tuberous sclerosis 2 (*TSC2*) (Figure 7C). The significance of this up-regulation remains to be determined, but is consistent with *AKT1(E17K)* suppression of *HER2*-driven tumorigenesis. Taken together, these data support a model in which *AKT1(E17K)* is not sufficient to promote signals that promote tumorigenesis because it attenuates RTK signaling through negative feedback control.

## DISCUSSION

In this study we generated the first genetically engineered mouse model for the oncogenic *AKT1(E17K)* somatic mutation in the mammary gland. This mutation has been detected in approximately 4-8% of breast tumors, with a strong association with ER expression. Although *AKT1(E17K)* can drive transformation of fibroblasts *in vitro* [7] and enhances the survival and migration of luminal breast epithelial cells [18], we find that expression of *AKT1(E17K)* in MCF10A cells does not strongly activate downstream signaling, nor does it enhance proliferation, consistent with previous findings [17]. Consistently, transgenic mice expressing *AKT1(E17K)* driven by the MMTV promoter develop hyperplastic lesions that do not progress to carcinoma. This hyperplasia is concomitant with an increase in ER expression, suggesting that ER activity may enable *AKT1(E17K)* to promote proliferation *in vivo*. Previous studies that have evaluated the functional significance of the *AKT1(E17K)* mutation *in vitro*, including our own, have been primarily performed in ER-negative cell lines. More recently, a study demonstrated that replacing the oncogenic *PIK3CA(E545K)* mutation with wild-type *PIK3CA* in MCF7 luminal breast cancer cells that are ER-positive in the context of *AKT1(E17K)* knock-in leads to increased transformation and xenograft tumor volume, demonstrating that *AKT1(E17K)* can function as an oncogene in the context of an ER-positive, luminal breast cancer [19]. Together with the observation that *AKT1(E17K)* expression leads to different phenotypes in mammary myoepithelial *versus* luminal cells [18], it is likely that this mutation is selected for in human breast cancer under specific genetic and cellular contexts that enable it to function effectively as an oncogene.

The finding that *AKT1(E17K)* suppresses *HER2*-mediated mammary tumorigenesis is in stark contrast with previous findings that have examined activated AKT1 transgenes in the same context. Specifically, both phospho-mimetic *AKT1(T308D/S473D)* and Myr-AKT1 transgenes accelerate *HER2*-driven mammary tumor formation [25, 32]. There are likely a number of reasons why *AKT1(E17K)* does not phenocopy artificially-activated AKT1 with respect to *HER2*-mediated tumor progression. First, both *AKT1(T308D/S473D)* and Myr-AKT1 have a quantitatively higher constitutive protein kinase activity that is not recapitulated by *AKT1(E17K)*. *AKT1(T308D/S473D)* is constitutively basally hyperactive and not subject to inactivation by dephosphorylation, and similarly Myr-AKT1 is constitutively membrane-localized and phosphorylated. Moreover, molecular dynamics simulations have revealed that the increased membrane association of the AKT1(E17K) mutant is due to rapid conformational changes in the PH domain that likely explain the pathological consequences attributed to this mutation [34]. Another likely explanation that accounts for the suppressed *HER2*-driven tumors in the context of *AKT1(E17K)* is feedback inhibition of RTK signaling. Several lines of evidence support this conclusion. For example, studies have shown that inhibition of Akt leads to up-regulation of several RTKs, including insulin receptor, IGF-1R and HER3 [35]. By extension, one would predict that in the converse situation, *AKT1(E17K)* would suppress RTK expression, and indeed we observe this correlation in both MMTV-tTA;*hAKT1(E17K)*;MMTV-*HER2* mice (Figure 6D) and human tumors harboring *AKT1(E17K)* (Figure 7B). Although Myr-AKT1 transgenic mice show decreased RTK expression [32], this hyperactive allele is sufficient to propagate downstream signaling to enhance phenotypes that control tumor progression, even in the presence of the negative feedback. By contrast, the relatively lower constitutive activity of AKT1(E17K) is not sufficient to promote downstream signaling (Figure 1A). Therefore, *AKT1(E17K)* suppresses HER2 signaling in a manner that is not overcome by its own protein kinase activity, ultimately leading to suppression of tumorigenesis.

Since *AKT1(E17K)* expression in the mouse mammary gland does not lead to tumor formation, this begs the question as to why this mutation is detected in 4-8% of breast cancer patients [7, 16]. One study revealed that the *AKT1(E17K)* somatic mutation occurs early in breast tumor progression, and therefore one can speculate that subsequent and additional genetic lesions are required for *AKT1(E17K)* to function as an oncogene in breast carcinoma [36]. Since *AKT1(E17K)* escapes feedback inhibition and displays sustained activation kinetics (Figure 2), it is likely that additional genetic lesions may potentiate its protein kinase activity. The nature of these genetic lesions remains to be determined, but are not likely to include oncogenic *PIK3CA* mutations or *PTEN* inactivation, which have been shown to be mutually exclusive with *AKT1(E17K)*, at least in breast tumors [16]. Based on the correlation of RTK suppression in the MMTV-tTA;*hAKT1(E17K)*;MMTV-*HER2* mice and in human *AKT1(E17K)* tumors, we also predict that cooperating mutations likely do not occur in signaling pathways that function through RTKs, particularly those in the EGFR family.

The findings presented in this study have implications for therapeutic intervention and development of targeted therapies for both RTKs as well as PI 3-K and Akt. There are presently numerous phase I and II clinical trials with a variety of allosteric and catalytic Akt small molecule inhibitors for therapeutic benefit in breast and other solid tumors [1, 37]. Since inhibition of Akt using catalytic inhibitors leads to the up-regulation of RTK signaling [35], and *AKT1(E17K)* tumors show suppressed levels and phosphorylation of RTKs, one implication is that breast cancer patients with *HER2* amplification would not benefit from treatment with Akt inhibitors. Instead, combination therapy with Akt inhibitors followed by RTK inhibition may be a more effective therapeutic strategy [38]. It is also important to note that the E17K somatic mutation has also been identified in *AKT2* in breast cancer, albeit at lower frequency, and also in *AKT3* in human melanoma. Whether the same mechanism of RTK suppression and escape of feedback inhibition applies to these mutants remains to determined. Regardless, these findings underscore the importance of understanding the mechanisms by which oncogenes escape feedback inhibition to lead to tumor initiation and progression.

## MATERIALS AND METHODS

### Cell lines

MCF10A cells were obtained from the American Type Culture Collection (ATCC) and authenticated using short tandem repeat (STR) profiling. Cells were maintained in DMEM/Ham's F12 supplemented with 5% equine serum (Gibco), 10 mg/mL insulin, 500 ng/mL hydrocortisone (Sigma-Aldrich), 20 ng/mL EGF (R&D Systems), and 100 ng/mL cholera toxin (Sigma-Aldrich). Cells were passaged for no more than 6 months and routinely assayed for mycoplasma contamination.

### Plasmids

The *AKT1(E17K)* mutation was generated by site-directed mutagenesis (Qiagen) from HA-*AKT1*/pcDNA3 (Addgene). For doxycycline-inducible overexpression of *AKT1* and *AKT1(E17K)*, HA-*AKT1*/pTRIPZ and HA-*AKT1(E17K)*/pTRIPZ were constructed. HA-*AKT1* and HA-*AKT1(E17K)* cDNA was amplified by PCR from HA-*AKT1*/pcDNA3 and HA- *AKT1(E17K)*/pcDNA3. The resulting PCR product was digested with restriction enzymes AgeI and ClaI, followed by insertion into the pTRIPZ lentiviral vector (Thermo Scientific).

### Immunoblotting

Cells were washed with PBS at 4°C and lysed in radioimmunoprecipitation assay buffer (1% NP-40, 0.5% sodium deoxycholate, 0.1% SDS, 150 mmol/L NaCl, 50 mmol/L Tris-HCl (pH 7.5), proteinase inhibitor cocktail, 50 nmol/L calyculin, 1 mmol/L sodium pyrophosphate, and 20 mmol/L sodium fluoride) for 15 minutes at 4°C. Cell extracts were pre-cleared by centrifugation at 13,000 rpm for 10 minutes at 4°C, and protein concentration was measured with the Bio-Rad DC protein assay. Lysates were then resolved on 10% acrylamide gels by SDS-PAGE and transferred electrophoretically to nitrocellulose membrane (Bio-Rad) at 100 V for 90 minutes. The blots were blocked in Tris-buffered saline (TBST) buffer (10 mmol/L Tris-HCl, pH 8, 150 mmol/L NaCl, and 0.2% Tween 20) containing 5% (w/v) nonfat dry milk for 1 hour, and then incubated with the specific primary antibody diluted in blocking buffer at 4°C overnight. Membranes were washed three times in TBST and incubated with HRP-conjugated secondary antibody for 1 hour at room temperature. Membranes were washed three times and developed using enhanced chemiluminescence substrate (EMD Millipore).

### *In vitro* protein kinase assays

MCF10A cells expressing HA-*AKT1* or HA-*AKT1(E17K)* were serum starved for 16 hours. HA-*AKT1* or HA-*AKT1(E17K)* were immunoprecipitated from cell extracts with an anti-HA antibody and incubated with 300 ng GSK-3 fusion protein peptide (Cell Signaling Technology) in the presence of 150 μmol/L cold ATP in a kinase buffer for 40 min at 30°C. The kinase reaction was terminated by the addition of SDS-PAGE sample buffer.

### Antibodies

All antibodies except the anti-HA and anti-p85 antibodies were purchased from Cell Signaling Technology. The anti-HA monoclonal antibody was purified from the 12CA5 hybridoma. The anti-p85 antibody has been described [39].

### Proliferation assays

MCF10A cells were seeded into 96-well plates at a density of 1500 cells per well in 100 μL medium. The medium was replaced with serum-free medium after 16 h. Cell viability was measured 0, 1, 2, and 3 days after the media change using the water soluble tetrazolium salt WST-1 assay (Clontech) according to the manufacturer's protocol.

### Generation of hAKT1(E17K) transgenic mice

HA-tagged *hAKT1(E17K)* (Addgene) was cloned into the Tetracycline-regulated (Tet-off) vector pTET Splice [40]. DNA was prepared for microinjection, performed at the Transgenic Animal Core Facility at Beth Israel Deaconess Medical Center under IACUC approved protocols. Resulting founder animals generated on an FVB background were genotyped using genomic DNA purified from a tail biopsy and performing PCR utilizing the following primer sequences: FW: 5′-CTGGAATTCATGTACCCATACGATGTTCCAG-3′ RV: 5′-CCT CTTCTTGAGGCCGTCAGCCACAGTCTGG-3′

### Validation of protein expression

HA-*hAKT1(E17K)* protein expression was confirmed by crossing animals positive for the transgene with VE-Cadherin-tTA driver mice [40]. Livers were collected from pups at P10 and screened for protein expression using immunoblot for HA (12CA5 Hybridoma) as previously described [41].

### Mammary specific transgene expression

*hAKT1(E17K)* transgenic mice that screened PCR positive were crossed with MMTV-tTA mice generated by Henninghausen et. al. as previously described [42], and the offspring were re-genotyped for the presence of the *hAKT1(E17K)* transgene as well as for MMTV-tTA using the primer sequences: FW: 5′-GACGCCTTAGCCATT AGAT-3′ RV: 5′-CAGTAGTAGGTGTTTCCCTTTCTT-3′. Double positive transgenic females were either maintained as virgins or were multi-paired. Double positive males were bred with MMTV-*HER2* homozygous females (Jackson Laboratories, #002376) and offspring were re-genotyped to confirm the presence of the transgenes.

### Mammary gland wholemount staining

Mammary fat pads were removed and placed on glass microslides and allowed to dry for 5 min, and then fixed overnight in Carnoy's fixative (60% ethanol, 30% chloroform, 10% acetic acid) at room temperature. After fixation, mammary glands were stained with Carmine alum.

### Immunohistochemistry

Mammary glands were removed and fixed in 10% NBF and processed for histology and evaluated by H&E staining at the Harvard Medical School Rodent Histopathology Core and the Beth Israel Deaconess Medical Center Histology Core. Immunohistochemistry on paraffin sections was performed for α-HA (12CA5 Hybridoma) utilizing a M.O.M kit (Vector labs, BMK-2202). Immunohistochemistry for α-ER (Millipore, #04-227), α-pAkt Ser473 (Cell Signaling Technology #4060), α-HER2 (Cell Signaling Technology #4290), α-pHER2 pTyr877 (Abcam #ab47262), α-HER3 (Cell Signaling Technology #12708), α-pHER3 pTyr1289 (Cell Signaling Technology #4791), α-EGFR (Cell Signaling Technology #4267), pEGFR pTyr1068 (Cell Signaling Technology #3777), α-CK8 (Developmental Studies Hybridoma Bank clone Troma-I), and α-CK14 (Biolegend PRB-155) was performed as follows: Briefly, sections were deparaffinized and rehydrated. Antigen unmasking was performed using target retrieval solution (DAKO #S2367). Endogenous peroxidases were blocked using 3% H_2_0_2_ and immunostaining performed using the Rabbit Vectastain ABC kit (Vector labs #PK-4001) and developed using a DAB peroxidase substrate kit (Vector labs #SK-4100).

## SUPPLEMENTARY FIGURES


