# Supplementary material for: Oncogenic AKT1(E17K) mutation induces mammary hyperplasia but prevents HER2-driven tumorigenesis
Source: Oncotarget. 2016 Mar 18;7(14):17301–13. doi: 10.18632/oncotarget.8191 (PMC4951213; doi:10.18632/oncotarget.8191)
Supplement: Supplementary file 1 [file oncotarget-07-17301-s001.pdf]

Oncogenic AKT1(E17K) mutation induces mammary hyperplasia but prevents HER2-driven tumorigenesis

Supplementary Material

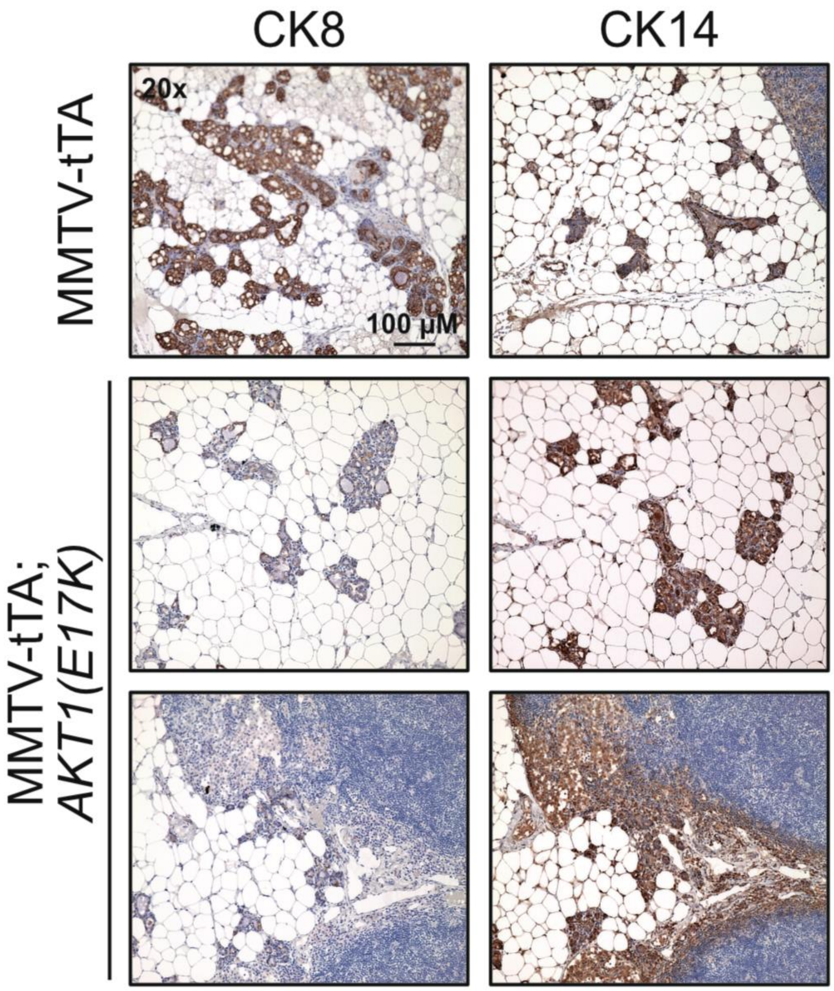

Supplementary Figure S1. MMTV-tTA;*hAKT1(E17K)* expression in the mammary epithelium induces mammary gland hyperplasia that results from an expansion of the myoepithelium. Immunostaining against CK8, a luminal marker, and CK14, a myoepithelial marker, was performed on paraffin sections of mammary glands from MMTV-tTA and MMTV-tTA;*hAKT1(E17K)* mice.

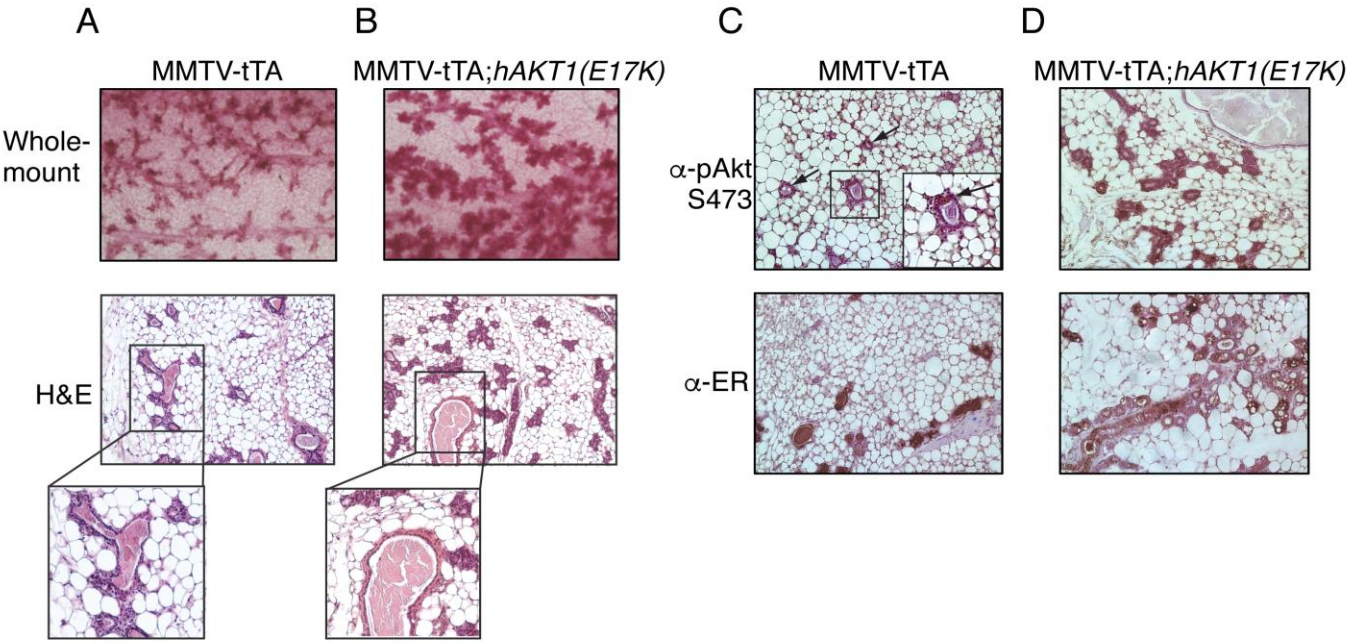

Supplementary Figure S2. Addition of an estrogen pellet does not result in mammary epithelial cell transformation or enhance hyperplasia. Multiparous MMTV-tTA;*hAKT1(E17K)* transgenic mice were exposed to a slow-release pellet of 17®-estradiol for 63 days. Wholemount analysis of mammary glands was performed from MMTV-tTA (A) or MMTV-tTA;*hAKT1(E17K)* (B) mice. Hematoxylin and eosin staining was also done on sections of paraffin embedded mammary glands from MMTV-tTA (A) or MMTV-tTA;*hAKT1(E17K)* (B) mice. Immunohistochemistry with 〈-pAkt Ser473 and 〈-ER antibodies was performed for paraffin embedded mammary glands from MMTV-tTA (C) or MMTV- tTA;*hAKT1(E17K)* (D) mice.
